# The Entropy Density Behavior across a Plane Shock Wave

**DOI:** 10.3390/e25060906

**Published:** 2023-06-07

**Authors:** Rosa M. Velasco, Francisco J. Uribe

**Affiliations:** Department of Physics, Universidad Autónoma Metropolitana, Av. San Rafael Atlixco 186, Iztapalapa, México City 09340, Mexico; paco@xanum.uam.mx

**Keywords:** entropy density, local equilibrium hypothesis, shock wave

## Abstract

Entropy density behavior poses many problems when we study non-equilibrium situations. In particular, the local equilibrium hypothesis (LEH) has played a very important role and is taken for granted in non-equilibrium problems, no matter how extreme they are. In this paper we would like to calculate the Boltzmann entropy balance equation for a plane shock wave and show its performance for Grad’s 13-moment approximation and the Navier–Stokes–Fourier equations. In fact, we calculate the correction for the LEH in Grad’s case and discuss its properties.

## 1. Introduction

Although the entropy concept has been considered for about 150 years, there are issues that have pervaded until the first quarter of the XXI century; a crucial one was presented succinctly by Hoover and Hoover in the preface to the first edition of their book entitled “*Time Reversibility, Computer Simulation, Algorithms, Chaos*” [1]:

Today a small army of physicists, chemists, mathematicians and engineers has joined forces for a renewed attack on a classical problem, the “irreversibility paradox”.

The irreversibility paradox is also known as Loschmidt’s paradox, since he pointed out that Newton’s laws are invariant under time reversal while, Boltzmann’s H function is not. Later on, Zermelo used Poincaré’s recurrence theorem to provide further criticisms of Boltzmann’s probabilistic interpretation of the second law, which led to a continuing exchange between Boltzmann and Zermelo [2,3]. The more recent discoveries (chaos, Lyapunov exponents, thermostats and fractals) have played a role in discussions of the irreversibility paradox in the XX and XXI centuries [1].

There is a comment by von Neumann to Shannon in relation to a question raised by the latter about what would be an appropriate term for a formula that he derived; the following answer by von Neumann to Shanon was communicated to Myron Tribus by Shannon [4]:

“You should call it entropy, for two reasons. In the first place your uncertainty function has been used in statistical mechanics under that name, so it already has a name. In the second place, and more important, no one really knows what entropy really is, so in a debate you will always have the advantage.”

The previous joke is important because it shows that entropy is not a simple concept. In fact, its meaning must be given within a clear context which settles the frame for its correct interpretation.

The concept of entropy may be attributed to Claussius (1879) [5], however, as it is usual for scientists to use the previous intuition of other authors. In the case of entropy, Carnot’s ideas [6] were essential to the development of the entropy concept, providing Clausius with relevant insights about it [7,8]. The origin of the concept by Carnot and Clausius was in the phenomenological field of classical thermodynamics, meaning that it is given in terms of macroscopic variables. In the second half of the XIX century, Boltzmann [9] adopted the nascent atomic hypothesis, an idea that at such time had detractors, to develop an expression for entropy which is engraved on his tombstone [7]

(1) $$S_{{}_{\;W}}=k_{B}\;\ln W,$$

where $S_{{}_{\;W}}$ is the entropy, $k_{B}$ is the Boltzmann constant and *W* the number of microstates compatible with the conditions of an isolated macroscopic system. Furthermore, he constructed the Boltzmann kinetic equation and defined his famous (H) functional in terms of the distribution function f(c,t), for homogeneous systems. It is simply generalized to consider the non-homogeneous case in which f(c,x,t), then

(2) H(x,t)=∫f(c,x,t)Lnf(c,x,t)dc,

where f(c,x,t)dcdx gives us the number of particles which at time *t* are in the volume element dcdx. It should be mentioned that the distribution function is now defined in the so called μ space (c,x) and it must be a solution of the Boltzmann equation. In the case of equilibrium, the distribution function becomes Maxwell’s velocity distribution [3,10], and it gives the negative of the equilibrium entropy for an ideal gas, and as a consequence, it is natural to define the local entropy as

(3) $$S_{H}\left(x,t\right)=-k_{B}H\left(x,t\right).$$

The entropy definition given in Equation (3) is valid for non-equilibrium situations as far as the H functional is well defined.

We do not pretend to give a review on the origin of the concept and its development; the interested reader will find critical insights in references [1,3,4,9,10,11,12,13] among others.

In this work our objective is more modest and amounts to a discussion of the entropy as given by Boltzmann’s H entropy $\left(S_{H}\right)$ for a shock wave and its relation with the local equilibrium hypothesis. As we will see, even in this simple case some problems arise.

The structure of this work is as follows. After this section we discuss the shock-wave problem in Section 2. Section 3 deals with the distribution function for Grad’s 13-moment approximation and the one provided by the Chapman–Enskog method at the Navier–Stokes–Fourier (NSF) level. In Section 4 we deal with the entropy density, and in Section 5 we provide numerical results for several profiles. We end by giving some final remarks in Section 6.

## 2. The Shock-Wave Structure

Let us consider a dilute gas in which a strong perturbation is produced so that a shock wave propagates with a constant velocity along the positive *x* direction. In the reference frame traveling with the shock wave we will have a stationary problem. Under these conditions, the usual mass, momentum and total energy conservation equations reduce to the case where the mass, linear momentum and total energy fluxes become constants. Then

(4) $$\begin{array}{c} \rho u=c_{1},\;\;\rho u^{2}+P_{xx}=c_{2}, \end{array}$$

(5) $$\begin{array}{c} \rho \left(\frac{u^{2}}{2}+e_{int}\right)u+P_{xx}u+q_{x}=c_{3}, \end{array}$$

where ρ is the mass density, *u* the velocity, $P_{xx}=p+p_{xx}$ is the xx component of the pressure tensor, *p* is the pressure, *T* the temperature, $p_{xx}$ the xx component of the viscous tensor, $e_{int}$ the internal energy density and $q_{x}$ the heat flux. The quantities $c_{1},\;c_{2},\;c_{3}$ are constants to be determined. We will consider an ideal monatomic gas in such a way that the pressure is related to the density and temperature through the ideal gas equation of state: $p=\rho \;k_{B}\;T$. In this case, the internal energy is given as $e_{int}=\frac{3}{2}\frac{k_{B}T}{m}$. The thermodynamic equilibrium points are characterized by $P_{xx}=p,\;q_{x}=0$, and we take the so-called upflow point as a reference $\left(\rho_{0},u_{0}\right)$, which corresponds to the cold and supersonic part of the shock. Now we define reduced variables as

(6) $$v=\frac{u}{u_{0}},\;\;\rho^{*}=\frac{\rho }{\rho_{0}},\;\;\tau =\frac{k_{B}T}{mu_{0}^{2}},\;\;s=\frac{x}{\lambda },\;\;\lambda =\frac{4}{3}\frac{\mu_{0}}{\rho_{0}u_{0}},\;\;\eta =\frac{\mu }{\mu_{0}},\;\;P=\frac{p_{xx}}{\rho_{0}u_{0}^{2}},\;\;Q=\frac{q_{x}}{\rho_{0}u_{0}^{3}},$$

where the subscript 0 means the values of the quantities evaluated at the upflow. Equations (4) and (5), written in terms of the reduced variables, are given as

(7) $$\rho^{*}v=1,\;P+\frac{\tau }{v}+v-1-\tau_{0}=0,\;vP+Q+\frac{1}{2}\left(5\tau -5\tau_{0}+v^{2}-1\right)=0;$$

their solution when P=0 and Q=0 are called the Rankine–Hugoniot jump conditions and determine the equilibrium points coordinates. One of them is the upflow, which is taken as the reference, and the downflow is characterized as the subsonic and hot part of the shock:

(8) $$\begin{array}{c} \mathrm{Upflow}:\;\;\;\;\;v_{0}=1,\;\;\tau =\tau_{0}, \end{array}$$

(9) $$\begin{array}{c} \mathrm{Downflow}:\;\;\;\;\;v_{1}=\frac{1+5\tau_{0}}{4},\;\tau_{1}=\frac{3+14\tau_{0}-5\tau_{0}^{2}}{16}, \end{array}$$

where $\tau_{0}=\frac{3}{5M^{2}}$ and $M=\frac{u_{0}}{c_{0}}$ is the Mach number and $c_{0}$ the speed of sound in the fluid calculated at upflow.

Now let us calculate the entropy density change occurring between the equilibrium points taking the upflow and downflow coordinates. Since we are dealing with an ideal gas, the entropy change is well defined between the upflow and downflow coordinates and we obtain

(10) $$\frac{m\Delta S}{k_{B}}=\frac{m(S_{down}-S_{up})}{k_{B}}=Ln\left[{\left(\frac{\tau_{1}}{\tau_{0}}\right)}^{3/2}\left(\frac{v_{1}}{v_{0}}\right)\right]\;>\;0.$$

As an immediate consequence, it is clear that we are dealing with an irreversible process occurring between the equilibrium points. This means that some dissipative processes take place and the shock wave must have a structure caused by them [14,15,16,17]. Now the problem turns out to be a search for the behavior of the dissipative effects present in the problem. They are represented by the viscous tensor and the heat flux due to the fact that both have already appeared in the conservation Equations (4) and (5). To go further, we recall that the system is a dilute gas, where the ideal gas equation of state and the internal energy written in terms of local variables are valid for the stationary shock wave.

Here we will have two lanes of research: First, the phenomenological scheme based on the linear irreversible thermodynamics, which starts with the fundamental entropy relation written for local variables. This means that the set of macroscopic variables describing the system satisfies the “local equilibrium hypothesis”(LEH). Then, the thermodynamic equilibrium relations are valid for local variables, the entropy density balance equation has the common entropy flux and the entropy production is the product of fluxes and thermodynamic forces [18].

The other scheme goes through the kinetic theory of gases based on the Boltzmann kinetic equation and the corresponding distribution function (df). In this case, the entropy density is directly related with the H Boltzmann functional as written in Equation (3) [9]. The application of the Maxwell transport equation allows for the writing of an entropy density balance equation, which in some particular cases is compatible with the LEH. Its compatibility depends on the approximation taken for the distribution function.

## 3. The Distribution Function

The kinetic theory approach to the study of non-equilibrium behavior for a dilute gas is based on the Boltzmann equation describing the distribution function f(c,x,t) [10,19]. The kinetic scheme is not so easy to develop due to its structure and the lack of detailed knowledge about the intermolecular interaction between particles. As a consequence, some approximate methods to deal with it have been studied. Here we will restrict ourselves to the Chapman–Enskog (CE) and Grad’s moment methods; the first one is a perturbative method where the Knudsen number defined as the ratio of the mean free path in the gas and the macroscopic lenght play a very important role. On the other hand, Grad’s method is a cumulant expansion in terms of the so called peculiar velocity C=c−u(x,t), where u(x,t) corresponds to the average velocity in the gas [20] and c is the atomic velocity. Both have their own limitations. The CE method is a perturbative one, and consequently the Knudsen number must be small (Kn≪1) to obtain a reasonable approximation. On the other hand, Grad’s method does not contain a smallness parameter; instead, a closure hypothesis is involved. Here we will consider the first order Kn number expansion in the CE method which drives to the Navier–Stokes–Fourier (NSF) constitutive equations. This means that we will take the viscous tensor and the heat flux proportional to the corresponding thermodynamic forces, i.e., the velocity and temperature gradients. In contrast, Grad’s method is taken with a 13-moments closure hypothesis.

The local Maxwell distribution function is taken to start the writing of an approximate solution of the Boltzmann equation:

(11) $$f^{\left(0\right)}=n{\left(\frac{m}{2\pi k_{B}T}\right)}^{3/2}\;exp\left(-\frac{mC^{2}}{2k_{B}T}\right),$$

where n(x,t) is the local number density so that ρ(x,t)=n(x,t)m, and T(x,t) is the local temperature and C=∥C∥. The first order (Kn) in the CE approximation allows us to write the df as

(12) $$f^{CE}=f^{\left(0\right)}\left[1+\frac{mp_{ij}}{2pk_{B}T}C_{i}C_{j}-\frac{mq_{i}}{pk_{B}T}C_{i}\left(1-\frac{mC^{2}}{5k_{B}T}\right)\right],\;\;\;q_{i}=-\kappa \nabla_{i}T,\;\;\;p_{ij}=-2\mu \bar{\left(\nabla_{i}u_{j}\right)},$$

where κ is the thermal conductivity and μ the shear viscosity, and they depend on the intermolecular potential. We recall that the bulk viscosity vanishes for a dilute gas and $\bar{\left(\nabla_{i}u_{j}\right)}$ means the traceless ij components of the deformation rate tensor.

The Grad’s distribution function is written up to the thirteen moments approximation, called (G13). In this case we will have a set of 13 coupled and nonlinear equations containing the whole set of variables:

(13) $$f^{G13}=f^{\left(0\right)}\left[1+\frac{mp_{ij}}{2pk_{B}T}C_{i}C_{j}-\frac{mq_{i}}{pk_{B}T}C_{i}\left(1-\frac{mC^{2}}{5k_{B}T}\right)\right],$$

where the heat flux and the viscous tensor must be determined according to their transport equations, obtained directly from the Boltzmann equation. Both dfs (12) and (13) share their structure in the sense that they represent deviations of the local Maxwellian df, although they are qualitatively different due to the fact that the CE df is closed according to the first order Kn number. G13 has an arbitrary closure in 13 moments, leaving the viscous tensor and the heat flux to be determined by the equations of motion. In the particular case of the plane shock wave traveling along the *x*-direction, they can be written as

(14) $$f=f^{\left(0\right)}\left[1+\frac{mp_{xx}}{2pk_{B}T}\left(C_{x}^{2}-\frac{1}{2}\left(c_{y}^{2}+c_{z}^{2}\right)\right)-\frac{mq_{x}}{pk_{B}T}C_{x}\left(1-\frac{mC^{2}}{5k_{B}T}\right)\right],$$

which can be written in terms of the dimensionless velocity components:

(15) $$C_{r1}=\frac{\left(c_{x}-u_{0}\right)}{u_{0}},\;c_{r2}=\frac{c_{y}}{u_{0}},\;c_{r3}=\frac{c_{z}}{u_{0}}.$$

Then, the dfs are written as

(16) $$f^{\left(0\right)}=\frac{n}{u_{0}^{3}}{\left(\frac{1}{2\pi \tau }\right)}^{3/2}\;exp\left(-\frac{C_{r}^{2}}{2\tau }\right),$$

(17) $$f=\frac{n}{u_{0}^{3}}{\left(\frac{1}{2\pi \tau }\right)}^{3/2}\;exp\left(-\frac{C_{r}^{2}}{2\tau }\right)\left[1+\frac{vP}{2\tau^{2}}\left(C_{r1}^{2}-\frac{1}{2}\left(c_{r2}^{2}+c_{r3}^{2}\right)\right)-\frac{vQC_{r1}}{\tau^{2}}\left(\frac{C_{r}^{2}}{5\tau }-1\right)\right],$$

where we have taken $\rho^{*}v=1$ according to (7) valid for a steady shock wave. The df in Equation (17) can be written as

(18) $$f=f^{\left(0\right)}\left(1+\phi \right),$$

where ϕ represents the deviation from the local Maxwellian df. It should be noticed that in the CE df, the deviation represented is a first order in the Kn number, so ϕ∼O(Kn), whereas in G13 there is not a smallness parameter.

All the kinetic calculations will be based on the df written as in Equation (18), hence we should study its behavior as a function of the velocity for some Mach number values. To proceed, we define the transversal speed $C_{rt}=\pm \sqrt{C_{r2}^{2}+C_{r3}^{2}}$ and we give some examples where we calculate the dfs for different Mach number values usually taken to calculate the shock-wave structure. First of all we should notice that the heat flux and the viscous tensor values must be given. However, they are functions of the dimensionless position *s* along the shock wave, which means that we must calculate them in some position across the shock. We have chosen the upflow, downflow and the center points. The center is the taken with the condition that the normalized density profile satisfies the relation $\frac{\rho \left(s\right)-\rho_{0}}{\rho_{1}-\rho_{0}}=\frac{1}{2}$. Furthermore, we need the corresponding values for P,Q, which are calculated with the shock-wave NSF solution for a given Mach number. It should be noted that the density profiles depend on the Prandtl number and the viscosity model taken to solve NSF equations. In particular, we have taken the soft sphere temperature dependence in such a way that $\eta \left(s\right)={\left(\frac{\tau (s)}{\tau_{0}}\right)}^{\sigma }$. For Argon, we have fitted the value of the viscosity index (σ) so that the solutions to the NSF equations reproduce the experimental normalized density profiles provided by Alsmeyer [21,22]. The previous results have shown that the NSF equations are in good agreement with Alsmeyer’s normalized density profiles if the viscosity is enhanced; this procedure holds for Mach numbers in the range (1.55≤M≤9) provided that the viscosity index is fitted for each Mach number [22]. From the fitted NSF numerical solution we have determined the values xx component of the viscous pressure tensor and the heat flux needed to evaluate the distribution function given by Equation (17). Notice that a similar procedure can in principle be carried out for Grad’s approximation, but only for Mach numbers lower than 1.65, since for higher Mach numbers, Grad’s approximation does not provide a shock-wave solution [20].

**Figure 1:**
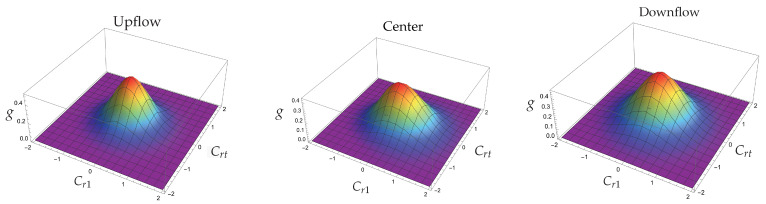
At the left, the distribution function corresponds to the cold part of the shock and is calculated using $v=1,\tau =\frac{240}{961},P=0$ and Q=0. For the graph in the middle we used the values v=0.720, τ=0.347, P=0.121 and Q=−0.095 that come from solving the NSF equations, using a viscosity index of value σ = 1.6, and are calculated at the position x=0; the origin is determined by requiring that the normalized density profile is 1/2 at x=0 [22]. The graph at the right corresponds to the hot part of the shock and is calculated using $v=\frac{2161}{3844},\tau =\frac{5711523}{14776336},P=0$ and Q=0.

Figure 1 shows the behavior of the reduced distribution function $g=u_{0}^{3}f/n_{0}$ as a function of the velocity components $C_{r1},\;C_{rt}$ for Ar at M=1.55.

**Figure 2:**
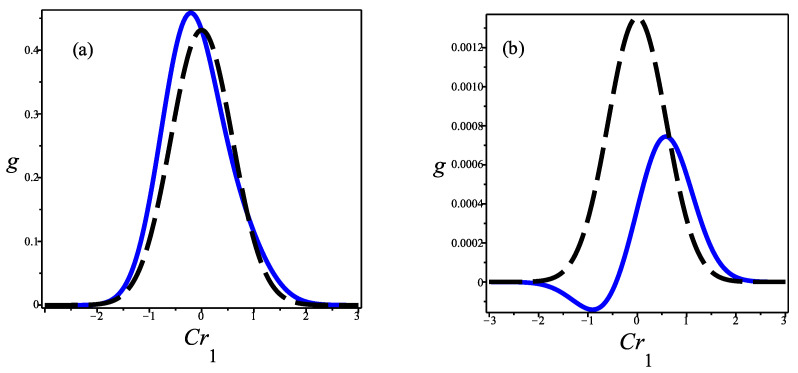
The intersection of the reduced distribution function $g\equiv u_{0}^{3}\;f/n_{0}$ *versus* $Cr_{1}$ with the planes (**a**) $Cr_{t}=0$ and (**b**) $Cr_{t}=2$. Here *f* is given by Equation (17), and the shock wave corresponds to M = 1.55 in Argon. The solid line corresponds to *g* and the dashed one to the local Maxwellian that is obtained from *g* by taking the fluxes equal to zero. We used the values v=0.720, τ=0.347, P=0.121 and Q=−0.095 that come from solving the NSF equations and are calculated, using a viscosity index of value σ = 1.6, at the position x=0; the origin is determined by requiring that the normalized density profile is 1/2 at x=0 [22].

The dfs calculated at the upflow and downflow are Gaussian dfs in the corresponding equilibrium points. The df named as center describes the situation when the normalized density equals 1/2. This point has been considered as representative of the region where the gradients in the normalized density and temperature profiles have their most important values. It has been observed that in such a region, the df may have negative values [23,24]. In Figure 1, negative values in the df are not apparent; however, in Figure 2 we provide two intersections of the distribution function with the planes $Cr_{t}=0$ and $Cr_{t}=2$; negative values of the distribution function are clearly exhibited in the last case.

**Figure 3:**
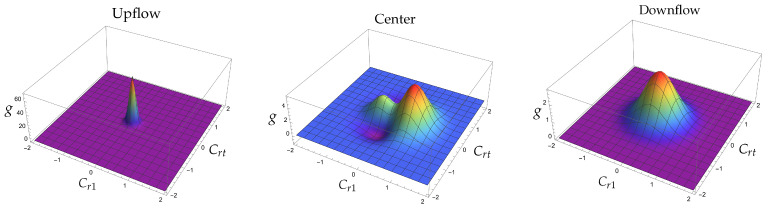
The reduced distribution function $g\equiv u_{0}^{3}\;f/n_{0}$ *versus* $C_{r1}$ and $C_{rt}\equiv +\sqrt{C_{r2}^{2}+C_{r3}^{2}}$, where *f* is given by Equation (16), for an M = 8 shock in Argon. At the left the distribution function corresponds to the cold part of the shock and is calculated using $v=1,\tau =\frac{3}{320},P=0$ and Q=0. For the graph in the middle we used the values v=0.422, τ=0.180, P=1.774 and Q=−0.790 that come from solving the NSF equations, using a viscosity index of value σ = 0.9, and are calculated at the position x=0; the origin is determined by requiring that the normalized density profile is 1/2 at x=0 [22]. The graph at the right corresponds to the hot part of the shock and is calculated using $v=\frac{67}{256},\tau =\frac{64119}{327680},P=0$ and Q=0.

For larger Mach numbers, the situation drastically changes, as shown in Figure 3.

On the other hand, we must notice that the calculations make sense only if the ϕ expansion is convergent, as usually it is argued when ϕ≪1.

## 4. The Entropy Density

The entropy density is defined as

(19) $$\rho \left(x,t\right)S\left(x,t\right)=-k_{B}\int_{-\infty }^{\infty }f\left(C,x,t\right)Ln\;\left(\frac{u_{0}^{3}f\left(C,x,t\right)}{n_{0}}\right)dC_{r},$$

where S(x,t) is the specific entropy. The calculations are performed with the CE and G13 dfs as written in Equation (18). Notice that the expression in (19) contains an adimensional quantity in the logarithmic function. This was taken to consider as a reference the numerical density $n_{0}$, which in the case of the shock wave will be the upflow value, as for all other variables in the system. Let us define $g\left(C,x,t\right)=\frac{u_{0}^{3}f\left(C,x,t\right)}{n_{0}},\;g^{\left(0\right)}\left(C,x,t\right)=\frac{u_{0}^{3}f\left(C,x,t\right)}{n_{0}}$ to shorten the notation.

(20) $$\begin{array}{ccc} & & \rho S=-k_{B}\int fLn\;gdC=-k_{B}\int f^{\left(0\right)}\left(1+\phi \right)\left[Ln\;g^{\left(0\right)}+Ln\;\left(1+\phi \right)\right]dC\\ & & =-k_{B}\int f^{\left(0\right)}\left[Ln\;g^{\left(0\right)}+\phi -\frac{\phi^{2}}{2}\right]dC-k_{B}\int f^{\left(0\right)}\phi \left[Ln\;g^{\left(0\right)}+\phi -\frac{1}{2}\phi^{2}\right]dC+\cdots \\ & & =\underset{\rho S^{loc}}{\underset{︸}{-k_{B}\int f^{\left(0\right)}Ln\;g^{\left(0\right)}}}dC\underset{\mathrm{vanishes}}{\underset{︸}{-k_{B}\int f^{\left(0\right)}\left(1+Ln\;g^{\left(0\right)}\right)\phi dC}}\underset{\mathrm{non}\;\mathrm{trivial}}{\underset{︸}{-\frac{k_{B}}{2}\int f^{\left(0\right)}\phi^{2}dC}}+\cdots \end{array}$$

The direct substitution of Equation (16) gives the expression for the local specific entropy, which in fact coincides with the equilibrium entropy where the equilibrium values are changed by their local counterparts,

(21) $$\frac{\rho S_{local}}{nk_{B}}=\frac{mS_{local}}{k_{B}}=\left[Ln\;\left(v\tau^{3/2}\right)+a\;\mathrm{constant}\right].$$

It should be noticed that the change of the local entropy between two points eliminates the constants, and we can then calculate the change in upflow–downflow in the shock wave. Furthermore, a different reference point can be chosen to measure the specific entropy; for example, we can take the entropy change between the upflow and any coordinate *x* before the downflow.

The second term in Equation (20) vanishes due to the compatibility conditions. The last term gives the first non trivial contribution consistent with the approximation $\phi^{2}$ we have considered. The corresponding calculation is direct, although somewhat cumbersome; then

(22) $$\frac{\rho \Delta S}{nk_{B}}=\frac{m\Delta S}{k_{B}}=\frac{\rho \Delta S_{local}}{nk_{B}}-\frac{v^{2}}{\tau^{2}}\left(\frac{3P^{2}}{8}+\frac{Q^{2}}{5\tau }\right)+...$$

Equation (22) shows that the entropy density has two completely different terms; the first one comes from the local contribution in the df. In contrast, the second part contains quadratic contributions in the dissipative effects. If we consider the CE expansion up to first order in the Kn number we notice that the local part is independent of the Knudsen number approximations. However, the terms containing the dissipative fluxes are second order Kn contributions. It is not the case when we consider the description in the Grad’s moments approximation; then the second term is the simplest non-trivial contribution to the entropy density.

The entropy flux will be defined as follows:

(23) $$J_{S}=-k_{B}\int CfLn\;\left(\frac{u_{0}^{3}f}{n_{0}}\right)dC,$$

where we only need the *x* component, which can be written as follows:

(24) $$\begin{array}{ccc} & & \frac{J_{Sx}}{k_{B}u_{0}^{4}}=-\int C_{rx}fLn\;gdC_{r}=-\int C_{rx}f^{\left(0\right)}\left(1+\phi \right)\left[Ln\;g^{\left(0\right)}+Ln\;\left(1+\phi \right)\right]dC_{r}\\ & & =-\int C_{rx}f^{\left(0\right)}\left[Ln\;g^{\left(0\right)}+\phi -\frac{\phi^{2}}{2}+...\right]dC_{r}=-\int C_{rx}f^{\left(0\right)}\phi \left[Ln\;g^{\left(0\right)}+\phi -\frac{1}{2}\phi^{2}+...\right]dC_{r}\\ & & \underset{︸}{=-\int C_{rx}\;f^{\left(0\right)}Ln\;g^{\left(0\right)}\;dC_{r}}\underset{︸}{-\int C_{rx}f^{\left(0\right)}Ln\;g^{\left(0\right)}\;\phi \;dC_{r}}\underset{︸}{-\frac{1}{2}\int C_{rx}f^{\left(0\right)}\phi^{2}dC_{r}}+...\\ & & \;\;\;\;vanishes\;\;\;\;\;\;\;\;\;\;\;\;\;\;\;\;non-trivial\;\;\;\;\;\;\;\;\;\;non-trivial \end{array}$$

Now we calculate the terms in Equation (24) and define the dimensionless entropy flux in the *x* direction giving the following result:

(25) $$\frac{J_{Sx}}{n_{0}k_{B}u_{0}}=j_{s}=\frac{Q}{\tau }\left(1-\frac{2}{5}\frac{vP}{\tau }+...\right),$$

valid up to the same approximation as the entropy density. It must be noted that Equation (22) is obtained from the local distribution function labeled as $f^{\left(0\right)}$, and the result is consistent with the entropy density in equilibrium evaluated with the local variables. Furthermore, due to the compatibility conditions, the entropy density can be calculated with *f* instead of $f^{\left(0\right)}$, and both results coincide. On the other hand, the first term in the entropy flux structure coincides with its usual expression in linear irreversible thermodynamics (LIT) [18] and it contains the heat flux over the temperature $\frac{Q}{\tau }$. This contribution is the first non-trivial term in the entropy flux and, in the CE approximation, is a first order Kn contribution. The second term corresponds to a correction which contains the product of dissipative fluxes, being a second order in the Knudsen number.

### 4.1. Navier–Stokes–Fourier (NSF)

To begin the analysis we will consider the first order Kn number in the df according to the CE method. In this case the constitutive equations for the dissipative constributions are given be the usual Navier–Newton and Fourier equations [10,19,23]. The NSF set of equations are then obtained directly by the direct substitution of the constitutive equations for the viscous tensor and the heat flow, as specified in Equation (12), all of them written in terms of the dimensionless variables defined in Equation (6). Then

(26) $$\begin{array}{c} \frac{\tau }{v}+v-\eta v^{\prime }=\tau_{0}+1, \end{array}$$

(27) $$\begin{array}{c} -v\eta v^{\prime }-\frac{15\eta }{8Pr}\tau^{\prime }+\frac{1}{2}\left(5\tau +v^{2}\right)=\frac{1}{2}\left(5\tau_{0}+1\right). \end{array}$$

where $v^{\prime },\;\tau^{\prime }$ mean the velocity and temperature derivatives with respect to *s*, $Pr=\frac{c_{p}\eta_{0}}{\kappa_{0}}=\frac{2}{3}$ is the Prandtl number and the reduced viscosity η is modeled through a power of the temperature:

(28) $$\eta =\frac{\mu }{\mu_{0}}={\left(\frac{\tau }{\tau_{0}}\right)}^{\sigma }.$$

The viscosity index (σ) can be determined by fitting experimental viscosity values or by using calculated viscosities obtained from formulas of the kinetic theory of gases with the interaction potential obtained from ab initio calculations.

Now we take the local equilibrium entropy density balance calculated as follows:

(29) $$\begin{array}{c} \frac{d}{dx}\left(\rho S_{local}u+J_{\;Sx}^{{}^{loc}}\right)=\frac{n_{0}k_{B}u_{0}}{\lambda }\frac{d}{ds}\left[Ln\;\left(v\;\tau^{3/2}\right)+\frac{Q}{\tau }\right]=\\ \frac{n_{0}k_{B}u_{0}}{\lambda }\left(-\frac{Q\tau^{\prime }}{\tau^{2}}+\frac{Q^{\prime }}{\tau }+\frac{\tau^{3/2}v^{\prime }+\left(3/2\right)v\sqrt{\tau }\tau^{\prime }}{v\tau^{3/2}}\right). \end{array}$$

According to the conservation laws valid in the shock wave (see Equation (7)), it is possible to write

(30) $$\begin{array}{c} Q^{\prime }=-\frac{3\tau^{\prime }}{2}-\left[1+\tau_{0}-v\right]v^{\prime },\;P=\frac{\left(1+\tau_{0}\right)v-v^{2}-\tau }{v},\\ \frac{d}{dx}\left(\rho S_{local}u+J_{\;Sx}^{{}^{loc}}\right)=\frac{n_{0}k_{B}u_{0}}{\lambda }\left(-Q\frac{\tau^{\prime }}{\tau^{2}}+\frac{\tau +v^{2}-v\left(1+\tau_{0}\right)}{v\tau }v^{\prime }\right)= \end{array}$$

(31) $$\begin{array}{c} \frac{n_{0}k_{B}u_{0}}{\lambda }\left(-\frac{\tau^{\prime }}{\tau^{2}}Q-\frac{v^{\prime }}{\tau }P\right).\;\;\;\;\;\;\;\;\;\;\;\;\;\;\;\;\;\;\;\; \end{array}$$

Therefore, the reduced entropy balance equation is

(32) $$\frac{d}{ds}\left(S_{local}^{*}+j_{{}_{\;Sx}}^{loc}\right)=\Sigma_{S}^{*}{|}_{local},$$

where

(33) $$S_{local}^{*}=\frac{m\;S_{local}}{k_{B}},\;j_{Sx}^{loc}=\frac{Q}{\tau },\;\Sigma_{S}^{*}{|}_{local}=\frac{\lambda }{n_{0}\;k_{B}\;u_{0}}\Sigma_{S}=-\frac{Q\;\tau^{\prime }}{\tau^{2}}-\frac{P\;v^{\prime }}{\tau }.$$

The entropy production density in its dimensionless expression is given as

(34) $$\Sigma_{S}^{*}{|}_{local}=-\frac{1}{\tau }\left(\frac{Q\tau^{\prime }}{\tau }+Pv^{\prime }\right).$$

We recall that the thermodynamic forces associated with the heat flux and viscous tensor are given as $X_{\tau }=\frac{d}{ds}\left(\frac{1}{\tau }\right),\;X_{v}=-\frac{1}{\tau }\frac{dv}{ds}$, respectively. The expression given in Equation (32) corresponds to the entropy density balance where the local equilibrium hypothesis is taken as it is in the usual linear irreversible thermodynamics approach [18]. In particular, the entropy production is the product of thermodynamic forces by fluxes.

(35) $$\Sigma_{S}^{*}{|}_{local}=X_{\tau }Q+X_{v}P.$$

To calculate the numerical values we need the solution for the local variables v(s),τ(s) and the corresponding fluxes P,Q. In this case we must take the NSF equations as written in Equations (26) and (27) to calculate the temperature and velocity profiles for a given Mach number and the viscosity index for a particular substance.

### 4.2. The Grad’s 13-Moment Approximation G13

Now we will work with G13 approximation, and as a first step we write the equations to be solved in this approximation. To do that we take the conservation equations, Equation (7) with the G13 equations, to determine the behavior of the viscous tensor and the heat flux, namely, [20,25],

(36) $$\begin{array}{c} \frac{3}{4}\frac{d}{ds}\left(vP\right)+\frac{2}{5}\frac{dQ}{ds}+P\left(\frac{\tau }{\eta v}+v^{\prime }\right)+\frac{\tau }{v}v^{\prime }=0, \end{array}$$

(37) $$\begin{array}{c} vQ^{\prime }+\left(\tau -vP\right)\frac{dP}{ds}+\frac{5\tau }{2v}\tau^{\prime }+\left[\frac{7}{2}\tau^{\prime }-v\left(\frac{d}{ds}\frac{\tau }{v}\right)\right]P+\left(\frac{16}{5}v^{\prime }+\frac{8\tau }{9\eta v}\right)Q=0. \end{array}$$

The set of Equations (7), (36) and (37) can be solved numerically to obtain the corresponding profiles with the same model for viscosity, as in the NSF case.

As a second step, the G13 df is taken to calculate the entropy density, which will contain the already calculated local part and some additional terms coming from the non-local contributions; both are shown in Equations (20)–(22). It should be noted that the non-local contributions are nonlinear terms in the viscous tensor and the heat flux. Furthermore, the entropy flux will have the local part and the non-local terms, as shown in Equation (25), where the non-local terms are bilinear in the viscous tensor and the heat flux. Now we will take the higher order terms, $O(\phi^{2})$, and calculate their derivative with respect to the dimensionless position; hence, using the results from the Appendix A, we obtain that the entropy balance equation is then written as follows:

(38) $$\begin{array}{c} \frac{d}{ds}\left(\rho S_{total}u+J_{{}_{Sx}}^{total}\right)=\frac{\lambda }{n_{0}\;k_{B}\;u_{0}}\{\frac{1}{\tau }\left(-\frac{Q\tau^{\prime }}{\tau^{2}}-\frac{Pv^{\prime }}{\tau }\right)\\ +\frac{1}{4\tau }\left(-\frac{7v^{\prime }}{5}+\frac{3v^{2}v^{\prime }}{\tau }+\frac{27v\tau^{\prime }}{5\tau }\right)P+\frac{1}{5\tau }\left(\frac{2\tau^{\prime }}{\tau }+\frac{4vv^{\prime }}{\tau }-\frac{2v^{\prime }}{v}+\frac{3\;v^{2}\;\tau^{\prime }}{\tau^{2}}\right)Q\\ +\frac{v}{4\tau^{2}}\left(\frac{3v\tau^{\prime }}{\tau }-\frac{7v^{\prime }}{5}\right)P^{2}+\frac{1}{5\tau^{3}}\left(\frac{3v^{2}\tau^{\prime }}{\tau }-2vv^{\prime }\right)Q^{2}+\frac{2}{5\tau^{2}}\left(-v^{\prime }+\frac{v^{2}v^{\prime }}{\tau }+\frac{2v\tau^{\prime }}{\tau }\right)PQ\}, \end{array}$$

where $\rho S_{total}u$ is given in Equation (22) and the entropy flux $J_{s}^{total}$ is written in Equation (25).

A close view of the entropy balance Equation (38) shows us that it contains the usual local part and several terms which have a different structure, in the sense that they are not the products of fluxes and thermodynamic forces as it happened in the NSF case (see Equation (35)). In fact, we know that the thermodynamics forces and their corresponding fluxes definitions are not unique due to the existence of Meixner transformations [26]. Hence, we wonder if it is possible to redefine the thermodynamic forces and recover the mentioned structure; however, it is easily seen that there are some kind of crossed effects which prevent such efforts. Furthermore, there are additional nonlinear contributions which make the problem worse.

## 5. The Local Equilibrium Hypothesis (LEH) and Beyond

In order to go a step further in the analysis of the calculations performed in the last section, we will specify as clearly as possible the content and implications of the local equilibrium hypothesis. First of all, it is important that it has been the cornerstone in the development of linear irreversible thermodynamics [18] and a lot of irreversible studies and generalizations [27,28,29,30,31,32,33,34]. It takes the classical thermodynamics relations to establish that the local variables describing a situation out of thermodynamic equilibrium can be written in the same way as they stand in thermodynamic equilibrium. This means that the equation of state, the caloric equation as well as the fundamental TdS relation are valid in local equilibrium. The line of thinking behind it focus on some situations which are not too far from true equilibrium, no matter that we do not have a quantitative criterion to be sure about such a fact. The NSF set of equations has kinetic support with the CE expansion in the Knudsen number Kn as we noticed before; the entropy density calculation must then be taken only with what we called the local term due to the fact that quadratic terms in the dissipative fluxes P,Q become of the order $Kn^{2}$. The entropy flux at order Kn is just proportional to Q/τ, and the entropy production up to the same order corresponds to the product of fluxes and the thermodynamic forces, as identified in the literature [18,35]. It should be mentioned that according to the LEH, the entropy production density must be positive definite for any position along the shock wave. This condition is completely fulfilled with the NSF set of equations. Sometimes it is taken to extract conclusions about the transport coefficients, shear viscosity, thermal conductivity, diffusion coefficients, etc., when they are needed. In this context we see that the NSF set of equations is completely consistent with the LEH up to the first order in the Knudsen number.

Concerning the G13 equations, we must recall that their kinetic support is given through an arbitrary closure hypothesis and they do not have a smallness parameter to give them a systematic way to characterize the kind of approximation they represent. With the calculations carried out before, we have a starting point to analyze the results. First of all, the calculations were performed with the $\phi^{2}$ contributions. The NSF calculations have the same structure; however, the Knudsen number order has given us a way to classify each term and neglect $Kn^{2}$ contributions. The lack of the smallness parameter in G13 avoids such a classification and we must consider the complete expression. Some terms are consistent with LEH, but some others are not. In this case we can say that we have non-local contributions which are not negligible. Obviously, the entropy production density has a different structure than the one consistent with LEH.

It should be pointed out that the G13 equations may have a phenomenological interpretation through the extended irreversible thermodynamic (EIT) description. EIT considers the fluxes as relevant and independent variables and as constituting the basis to write a generalization of the TdS fundamental relation [34]. The steps followed by EIT drive an entropy production with the same structure as we have found for the G13 set of equations [36]. This means that the LEH is not obeyed in the EIT formulation. A similar quotation can be made for Burnett equations, which rise as an approximate solution for the Boltzmann equation at the second order in the Kn number [10]. The Burnett’s equation’s origin is completely kinetic and derived from the CE method; however they have a thermodynamic context, as shown in Reference [37]. In both cases, G13 and Burnett, among other sets of equations, are not compatible with LEH, although they correspond to a thermodynamic context.

The numerical results will be given us a clear idea about the relevance of the non-local contributions.

## 6. Numerical Results

Let us study the performance of NSF and G13 in relation to the entropy density behavior. In order to compare the models the normalized variables are defined as follows:

(39) $$\rho_{n}\left(s\right)=\frac{1/v\left(s\right)-1/v_{0}}{1/v_{1}-1/v_{0}},\;\tau_{n}\left(s\right)=\frac{\tau \left(s\right)-\tau_{0}}{\tau_{1}-\tau_{0}}.$$

**Figure 4:**
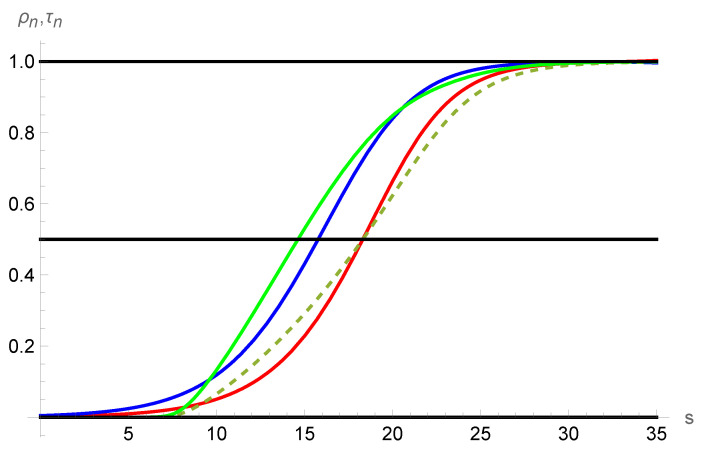
Normalized density and normalized temperature profiles for He at M=1.59 and the viscosity index σ=0.6716. The normalized density according NSF is the red line and the green dashed line gives the G13 profile. The blue line corresponds to NSF normalized temperature and the green solid line to the G13 model. The black central line indicates the values 1/2 for the normalized density, as usual in the literature.

Figure 4 shows the normalized and temperature profiles for He at M=1.59, and to reduce the variables we have taken the following:

(40) $$\begin{array}{ccc} u_{0} & = & 1183.3884\;\frac{\mathrm{mt}}{\sec},\;\rho_{0}=1.93\times 10^{-8}\;\frac{gr}{{\mathrm{cm}}^{3}},\;k_{B}=1.380649\times 10^{-23}\;\frac{J}{K},\\ m & = & 6.6465\times 10^{-27}\;\mathrm{kg},\;\mu_{0}=13.0310\times 10^{-6}\;\mathrm{Pa}\cdot s\;\Rightarrow \\ \lambda & \approx & 0.7607\;\mathrm{mm},\;\tau_{0}=\frac{3}{5\;M^{2}}\Rightarrow \tau_{0}=2000/8427\;\mathrm{for}\;M=1.59. \end{array}$$

In addition, we used the soft sphere model, given by Equation (28), to fit the values of the transport coefficients of ${}^{4}$He reported by Hurly [38] using ab initio values for the interatomic potential. The resulting value for the viscosity index, obtained from the fit, is σ=0.6716 [25]. With the previous information, the solutions to the NSF differential equations, given by Equations (26) and (27), or to Grad’s equations, given by Equations (36) and (37), were determined by electing the normalized density profile with the value 1/2 for s≈18.3 [25,39].

**Figure 5:**
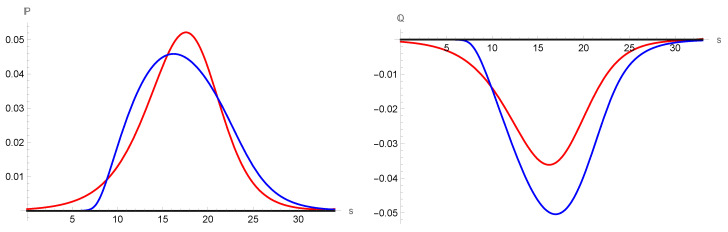
Comparison between the viscous tensor and heat flux profiles for He at M=1.59. Red solid lines correspond to the NSF model and the blue solid lines to the G13 approximation.

The normalized density and temperature profiles and their comparison with experimental data for G13 and NSF have been discussed in the literature [25,39], as shown in Figure 4. The results represented here are shown to demonstrate that they have the usual behavior in both NSF and G13 models. The viscous tensor and the heat flux are shown in Figure 5 for both models. It is clear that they vanish in the equilibrium points, as it should be.

**Figure 6:**
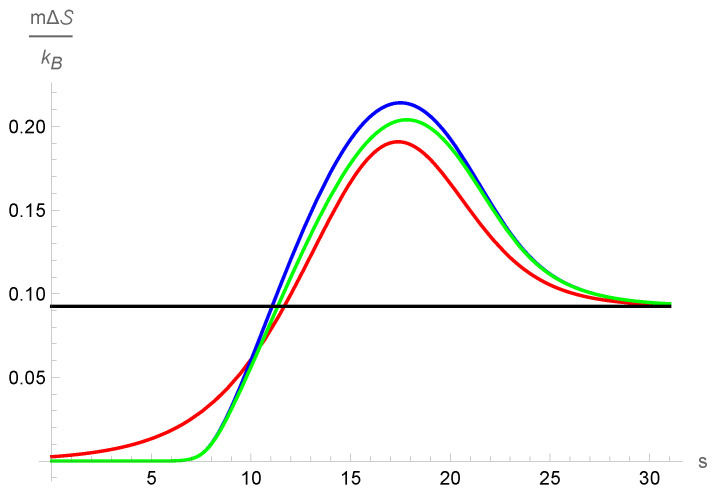
The entropy change profile according to the NSF (red line), local contribution to the entropy change according to the Grad-13 approximation (blue line) and the total entropy change (green line) calculated with the G13 profiles.

Figure 6 shows the entropy density change profile in the cases corresponding to the NSF local expression; the local contribution in G13 model and the complete entropy density change for the G13 case are calculated with the solution for the G13 equations. The comparison between the local and the total entropy change calculated with the G13 model solutions shows how relevant the non-local terms become. It shows in a clear way that the non-local contributions in the G13 approximation are not negligible. A conspicuous feature of the local entropy density profile is that it exhibits a maximum, as shown in Figure 6. The entropy discussed in the work by Margolin et al. [17] is monotonic, which is expected outside of equilibrium according to some researchers. However, the results of numerically solving the Boltzmann equation by Malkov et al. [40] report non-monotonic entropy profiles. For other theories in which the entropy in a shock wave is discussed, the interested reader is referred to the relevant bibliography [41,42].

**Figure 7:**
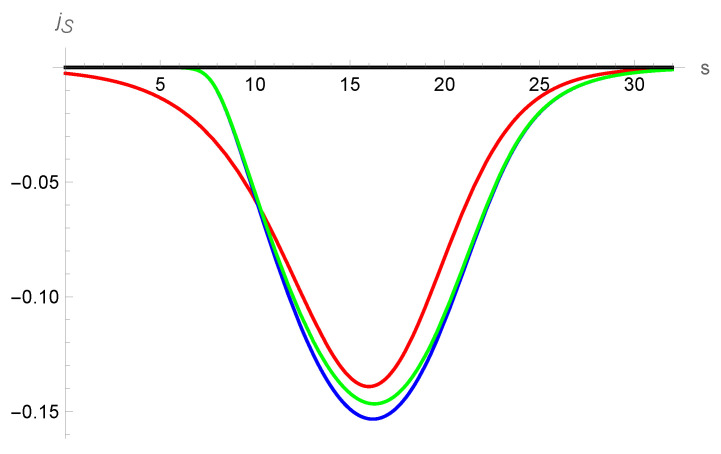
Entropy density flux in the NSF and Grad-13 approximation. The red solid line corresponds to NSF, the blue solid line corresponds to the local contribution according to the G13 case and the green line gives the total entropy density flux as calculated with the G13 profiles.

In Figure 7, the usual entropy flux for the NSF model is shown in contrast with the local contribution in the G13 case where a big difference is evident. In this case, the non-local contribution in the G13 approximation becomes the leading part.

**Figure 8:**
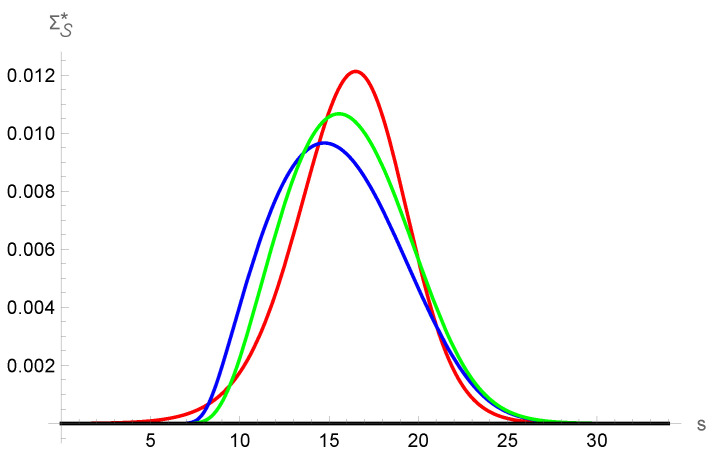
Entropy density production according to NSF and G13 models. The red solid line is the NSF profile, the blue solid line corresponds to the local contribution according to the G13 approximation and the green line gives the total entropy production calculated with the G13 profiles.

The entropy production is presented and compared in Figure 8, where it is shown that the non-local contribution plays a very significative role in the total entropy production. In this case, it is seen that its difference with the total entropy production is somewhat small.

## 7. Concluding Remarks

There is no doubt about the entropy concept difficulties, much of them caused because an incomplete context is given when it is applied to a particular problem. Even more, the local equilibrium hypothesis must be applied carefully, since it has somewhat blurry limitations. In particular, sometimes it is extended indiscriminately, taking as a basis the second law of thermodynamics for an isolated system, without questioning its validity under the particular conditions imposed by the problem. The work presented here has tried to show explicitly the contrast between the entropy calculations based on the NSF and the Grad13 models, within the frame of their application to study the shock-wave structure in dilute gases. The NSF model is consistent with the LEH, and the Grad13 moments do not perform in the same way. In fact, the calculation shown allows a quantitative estimate of how different the entropy production is in this case. The differences come from the numerical solutions for the variables (v(s),τ(s)) in each model; however, the difference in the structure is more important, as it is shown in Equation (38). In particular, the last three terms are second order in the fluxes, and due to the lack of a smallness parameter, their order of magnitude is not known. The entropy production in the G13 model does not have the structure required by the LHE. Moreover, its non-local contributions cannot be neglected, at least in this particular example. It must be said that Grad’s moment method is just one model to show the problem set in this work; for example, the Burnett set constitutes another set which is not consistent with LEH.

It should be noted that the shock-wave problem with the NSF or G13 equations can be solved independently of the definition of entropy used. For a glimpse of the vast literature available on shock waves in dilute gases, the reader can refer to the bibliography cited in reference [43], which also provides a few references to shock waves in dense gases. For the latter case, the interested reader may take a look at chapter 6 of the book by Hoover and Hoover [1].

## Data Availability

Not applicable.

## Appendix A

This appendix is devoted to some details concerning the non-local contributions to the entropy production in the G13 model. First of all, we write the non-local contributions in the entropy density and the entropy flux:

(A1) $$\begin{array}{c} \frac{\rho \Delta S}{nk_{B}}{|}_{non-local}=-\frac{v^{2}}{\tau^{2}}\left(\frac{3P^{2}}{8}+\frac{Q^{2}}{5\tau }+...\right) \end{array}$$

(A2) $$\begin{array}{c} j_{S}{|}_{non-local}=-\frac{2}{5}\frac{vPQ}{\tau^{2}}+... \end{array}$$

whose derivatives are

(A3) $$\frac{d}{ds}{\Delta S}_{non-local}^{*}=-\frac{3v^{2}PP^{\prime }}{4\tau^{2}}-\frac{2v^{2}QQ^{\prime }}{5\tau^{3}}+\left(-\frac{2vv^{\prime }}{5\tau^{3}}+\frac{3v^{2}\tau^{\prime }}{5\tau^{4}}\right)Q^{2}+\left(-\frac{3vv^{\prime }}{4\tau^{2}}+\frac{3v^{2}\tau^{\prime }}{4\tau^{3}}\right)P^{2},$$

(A4) $$\frac{d}{ds}j_{S}{|}_{non-local}=-\frac{2v}{5\tau^{2}}QP^{\prime }-\frac{2v}{5\tau^{2}}PQ^{\prime }-\left(\frac{2v^{\prime }}{5\tau^{2}}-\frac{4v\tau^{\prime }}{5\tau^{3}}\right)PQ.$$

We then obtain that the non–local entropy production is given by

(A5) $$\begin{array}{c} \Sigma_{S}^{*}{|}_{non-local}=-\left(\frac{2v}{5\tau^{2}}Q+\frac{3v^{2}}{4\tau^{2}}P\right)P^{\prime }-\left(\frac{2v^{2}}{5\tau^{3}}Q+\frac{2v}{5\tau^{2}}P\right)Q^{\prime }-\\ \left(\frac{2vv^{\prime }}{5\tau^{3}}-\frac{3v^{2}\tau^{\prime }}{5\tau^{4}}\right)Q^{2}+\left(-\frac{3vv^{\prime }}{4\tau^{2}}+\frac{3v^{2}\tau^{\prime }}{4\tau^{3}}\right)P^{2}+\left(-\frac{2v^{\prime }}{5\tau^{2}}+\frac{4v\tau^{\prime }}{5\tau^{3}}\right)PQ. \end{array}$$

Now we introduce the viscous tensor and heat flux derivatives taken from the conservation Equation (7) written as

(A6) $$P^{\prime }=\left(\frac{\tau }{v^{2}}-1\right)v^{\prime }-\frac{\tau^{\prime }}{v},\;\;Q^{\prime }=-\frac{3\tau^{\prime }}{2}-\left(P+\frac{\tau }{v}\right)v^{\prime },$$

so that

(A7) $$\begin{array}{c} \Sigma_{S}^{*}{|}_{non-local}=\frac{3P^{2}v^{2}\tau^{\prime }}{4\tau^{3}}-\frac{7P^{2}vv^{\prime }}{20\tau^{2}}-\frac{2PQv^{\prime }}{5\tau^{2}}+\frac{2PQv^{2}v^{\prime }}{5\tau^{3}}+\frac{4PQv\tau^{\prime }}{5\tau^{3}}-\frac{7Pv^{\prime }}{20\tau }\\ +\frac{3Pv^{2}v^{\prime }}{4\tau^{2}}+\frac{27Pv\tau^{\prime }}{20\tau^{2}}+\frac{3Q^{2}v^{2}\tau^{\prime }}{5\tau^{4}}-\frac{2Q^{2}vv^{\prime }}{5\tau^{3}}+\frac{2Q\tau^{\prime }}{5\tau^{2}}+\frac{3Q\tau^{\prime }}{5\tau^{3}}+\frac{4Qvv^{\prime }}{5\tau^{2}}-\frac{2Qv^{\prime }}{5\tau v}. \end{array}$$

Equation (A7) can then be simplified:

(A8) $$\begin{array}{c} \Sigma^{*}{|}_{non-local}=\frac{v}{4\tau^{2}}\left(\frac{3v\tau^{\prime }}{\tau }-\frac{7v^{\prime }}{5}\right)P^{2}+\frac{1}{5\tau^{3}}\left(\frac{3v^{2}\tau^{\prime }}{\tau }-2vv^{\prime }\right)Q^{2}+\\ \frac{2}{5\tau^{2}}\left(-v^{\prime }+\frac{v^{2}v^{\prime }}{\tau }+\frac{2v\tau^{\prime }}{\tau }\right)PQ+\frac{1}{4\tau }\left(-\frac{7v^{\prime }}{5}+\frac{3v^{2}v^{\prime }}{\tau }+\frac{27v\tau^{\prime }}{5\tau }\right)P+\\ \frac{1}{5\tau }\left(\frac{2\tau^{\prime }}{\tau }+\frac{4vv^{\prime }}{\tau }-\frac{2v^{\prime }}{v}+\frac{3\;v^{2}\;\tau^{\prime }}{\tau^{2}}\right)Q. \end{array}$$

## References

[1] Hoover W.G., Hoover C.G. (2012). Time Reversibility, Computer Simulation, Algorithms, Chaos.

[2] Steckline V.S. (1983). Zermelo, Boltzmann, and the recurrence paradox. Am. J. Phys..

[3] Brush S.G. (2003). The Kinetic Theory of Gases, an Anthology of Classic Papers with Historical Commentary.

[4] Tribus M., McIrvine E.C. (1971). Energy and information. Sci. Am..

[5] Clausius R. (1879). The Mechanical Theory of Heat.

[6] Carnot S. (1824). Reflexions sur la Puissance du feu et sur les Machines Propres à Developper Cette Puissance.

[7] Greven A., Keller G., Warnecke G., Greven A., Keller G., Warnecke G. (2003). Introduction. Entropy.

[8] Müller I., Greven A., Keller G., Warnecke G. (2003). Entropy: A subtle Concept in Thermodynamics. Entropy.

[9] Boltzmann L. (1964). Lectures on Gas Theory.

[10] Chapman S., Cowling T.G. (1970). The Mathematical Theory of Non-Uniform Gases.

[11] Greven A., Keller G., Warnecke G. (2003). Entropy.

[12] Singh M.S., O’neill M.E. (2022). The climate system and the second law of thermodynamics. Rev. Mod. Phys..

[13] Grandy W.T. (2008). Entropy and the Time Evolution of Macroscopic Systems.

[14] Zel’dovich Y.B., Yu P.R. (2002). Physics of Shock Waves and High—Temperature Hydrodynamic Phenomena.

[15] Serrin J., Whang Y.C. (1961). On the Entropy Change Through a Shock Layer. J. Aeorsp. Sci..

[16] Morduchow M., Libby P.A. (1949). On a complete solution of the one-dimensional flow equations of a viscous, heat conducting compressible gas. J. Aeronat. Sci..

[17] Margolin L.G., Reisner J.M., Jordan P.M. (2017). Entropy in self-similar shock profiles. Int. J. Non-Linear Mech..

[18] De Groot S.R., Mazur P. (1984). Non-Equilibrium Thermodynamics.

[19] Kremer G.M. (2010). An Introduction to the Boltzmann Equation and Transport Processes in Gases.

[20] Grad H. (1952). The profile of a steady plane shock wave. Commun. Pure Appl. Math..

[21] Alsmeyer H. (1976). Density profiles in argon and nitrogen shock waves measured by the absorption of an electron beam. J. Fluid Mech..

[22] Uribe F.J., Velasco R.M. (2018). Shock-wave structure based on the Navier–Stokes–Fourier equations. Phys. Rev. E.

[23] Struchtrup H. (2005). Macrocopic Transport Equations for Rarefied Gas Flows. Approximation Methods in Kinetic Theory.

[24] Velasco R.M., García–Colín L.S., Uribe F.J. (2011). Entropy Production: Its Role in Non-Equilibrium Thermodynamics. Entropy.

[25] Uribe F.J., Velasco R.M. (2022). Nonlinear transport coefficients from Grad’s 13–moment approximation. Meccanica.

[26] Piña E. (1979). On the De Donder–Meixner Transformations in Non-equilibrium Thermodynamics. Physica A.

[27] Truesdell C. (1984). Rational Thermodynamics.

[28] García–Colín L.S., Uribe F.J. (1991). Extended Irreversible Thermodynamics Beyond the Linear Regime: A Critical Overview. J. Non-Equilib. Thermodyn..

[29] Eu B.C. (1992). Kinetic Theory and Irreversible Thermodynamics.

[30] Maugin A.M., Muschik W. (1994). Thermodynamics with Internal Variables Part I. General Concepts. J. Non-Equilib. Thermodyn..

[31] Müller I., Ruggeri T. (1998). Rational Extended Thermodynamics.

[32] Öttinger H.C. (2005). Beyond Equilibrium Thermodynamics.

[33] Muschick W. (2007). Why so many “schools” of thermodynamics?. Forsch Ingenieurwes.

[34] Jou D., Casas-Vázquez J., Lebon G. (2010). Extended Irreversible Thermodynamics.

[35] Landau L.D., Lifshitz E.M. (1986). Fluid Mechanics.

[36] Velasco R.M., García-Colín L.S. (1992). The kinetic foundations of Extended Irreversible Thermodynamics revisited. J. Stat. Phys..

[37] García-Colín L.S., Velasco R.M., Uribe F.J. (2008). Beyond the Navier-Stokes equations: Burnett Hydrodynamics. Phys. Rep..

[38] Hurly J.B., Mehl J.B. (2007). 4He Thermophysical properties: New ab initio calculations. J. Res. Natl. Stand. Technol..

[39] Velasco R.M., Uribe F.J. (2021). A study on the Holian conjecture and Linear Irreversible Thermodynamics for shock–wave structure. Wave Motion.

[40] Malkov E.A., Bondar Y.A., Kokhanchick A.A., Poleshkin S.O., Ivanov M.S. (2015). High-accuracy deterministic solution of the Boltzmann equation for the shock wave structure. Shock Waves.

[41] Jou D., Pavón D. (1991). Nonlocal and nonlinear effects in shock waves. Phys. Rev. A.

[42] Al-Ghoul M., Eu B.C. (1997). Generalized hydrodynamics and shock waves. Phys. Rev. E.

[43] Uribe F.J. (2016). Shock waves: The Maxwell-Cattaneo case. Phys. Rev. E.

